# Thymoproteasomes produce unique peptide motifs for positive selection of CD8^+^ T cells

**DOI:** 10.1038/ncomms8484

**Published:** 2015-06-23

**Authors:** Katsuhiro Sasaki, Kensuke Takada, Yuki Ohte, Hiroyuki Kondo, Hiroyuki Sorimachi, Keiji Tanaka, Yousuke Takahama, Shigeo Murata

**Affiliations:** 1 Laboratory of Protein Metabolism, Graduate School of Pharmaceutical Sciences, The University of Tokyo, Tokyo 113-0033, Japan; 2 Division of Experimental Immunology, Institute for Genome Research, University of Tokushima, Tokushima 770-8503, Japan; 3 Calpain Project, Department of Advanced Science for Biomolecules, Tokyo Metropolitan Institute of Medical Science, Tokyo 156-8506, Japan; 4 Laboratory of Protein Metabolism, Tokyo Metropolitan Institute of Medical Science, Tokyo 156-8506, Japan

## Abstract

Positive selection in the thymus provides low-affinity T-cell receptor (TCR) engagement to support the development of potentially useful self-major histocompatibility complex class I (MHC-I)-restricted T cells. Optimal positive selection of CD8^+^ T cells requires cortical thymic epithelial cells that express β5t-containing thymoproteasomes (tCPs). However, how tCPs govern positive selection is unclear. Here we show that the tCPs produce unique cleavage motifs in digested peptides and in MHC-I-associated peptides. Interestingly, MHC-I-associated peptides carrying these tCP-dependent motifs are enriched with low-affinity TCR ligands that efficiently induce the positive selection of functionally competent CD8^+^ T cells in antigen-specific TCR-transgenic models. These results suggest that tCPs contribute to the positive selection of CD8^+^ T cells by preferentially producing low-affinity TCR ligand peptides.

## Abstract (web summary)

Proteasomes digest intracellular proteins into peptides that are then presented to lymphocytes as antigens. Here the authors show that a thymic epithelium-specific proteasome subunit cuts model proteins in a pattern favouring their weak binding to T cell receptor, and thus T cell positive selection.

## Graphical abstract

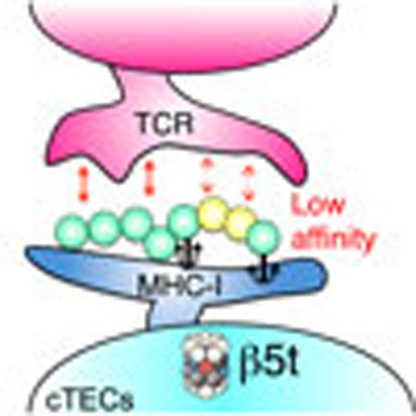

The proteasome is a proteinase complex that plays a pivotal role in protein homeostasis through degradation of regulatory and aberrant proteins tagged with ubiquitin in eukaryotic cells^1^,^2^. The catalytic core of proteasomes (core particle; CP) consists of 28 subunits, α_1–7_β_1–7_β_1–7_α_1–7_. Three of these subunits, β1, β2 and β5, have catalytic activity—caspase-like, trypsin-like and chymotrypsin-like, respectively—which cleaves peptide bonds after acidic, basic and hydrophobic residues, respectively. β1, β2 and β5 perform the catalytic function in ubiquitously expressed, constitutive CPs (cCPs)^3^,^4^,^5^.

Proteasomes digest proteins into short peptides, which serve as a principal source of antigenic peptides presented by major histocompatibility complex class I (MHC-I) molecules in vertebrates^6^,^7^,^8^. A small fraction of the peptides are conveyed into the endoplasmic reticulum, where their N termini are trimmed by aminopeptidases so that they fit exactly into the MHC-I grooves. Resultant MHC-I–peptide complexes are presented on the cell surface and recognized by T-cell receptors (TCRs) of CD8^+^ cytotoxic T cells (CTLs). Cells presenting only self-peptides are not attacked by CTLs, whereas cells presenting foreign or non-self antigens elicit a CTL response and are eliminated. Thus, the proteasome plays an essential role in self-non-self discrimination mediated by MHC-I in adaptive immunity.

Besides cCPs, two types of CP have been identified. One is the immunoproteasome (iCP), which incorporates β1i, β2i and β5i as catalytic subunits in place of β1, β2 and β5 of the cCP^9^,^10^,^11^,^12^. The iCP is expressed constitutively in haematopoietic cells and in cells stimulated with interferon-γ (IFN-γ). iCPs produce antigenic peptides for MHC-I more efficiently than cCPs and promote elimination of virus-infected cells by CD8^+^ T cells. The other is the thymoproteasome, or tCP, which contains β5t in place of β5i/β5 along with β1i and β2i and is exclusively expressed in cortical thymic epithelial cells (cTECs)^13^,^14^,^15^.

The catalytic properties of β5t seem quite different from those of β5i/β5, since there are marked differences between β5t and β5i/β5 in the substrate-binding pockets that determine the C terminus of a processed peptide^13^. The pockets of β5i/β5 are mostly composed of hydrophobic amino acids and thus elicit chymotrypsin-like activities, whereas the pocket of β5t is mostly composed of hydrophilic amino acids to which hydrophobic residues of peptides should be difficult to access. Indeed, incorporation of β5t into CPs reduced the chymotrypsin-like activity that cleaves peptide bonds after hydrophobic amino acids^13^.

The importance of the unique catalytic activity of tCPs was demonstrated by the analysis of mice deficient in β5t. *β5t*^−/−^ mice exhibited a severe defect in the positive selection of MHC-I-restricted CD8^+^ T cells in the thymus, even though thymus architecture and MHC-I expression on cTECs were normal^13^,^14^. β5i was compensatively expressed in *β5t*^−/−^ cTECs, where the iCP replaced the tCP^14^. It is widely accepted that the affinity between TCR and the MHC-I–peptide complex on cTECs determines the fate of developing thymocytes^16^,^17^,^18^,^19^. However, whether tCPs contribute to the production of MHC-I-associated peptides that carry the affinity suitable for the positive selection of CD8^+^ T cells is unclear.

Here we show that the tCPs contribute to the positive selection of CD8^+^ T cells by preferentially producing TCR ligand peptides that are efficient for inducing positive selection. We found that tCPs produce unique cleavage motifs in digested peptides and in MHC-I-associated peptides. Furthermore, MHC-I-associated peptides carrying these tCP-dependent motifs are enriched with low-affinity TCR ligands that efficiently induce the positive selection of functionally competent CD8^+^ T cells in antigen-specific TCR-transgenic models. Our results demonstrate that the unique catalytic activity of tCPs is a key mechanism underlying the positive selection of CD8^+^ T cells.

## Results

### Reconstitution of the tCP in cells

**Figure 1:**
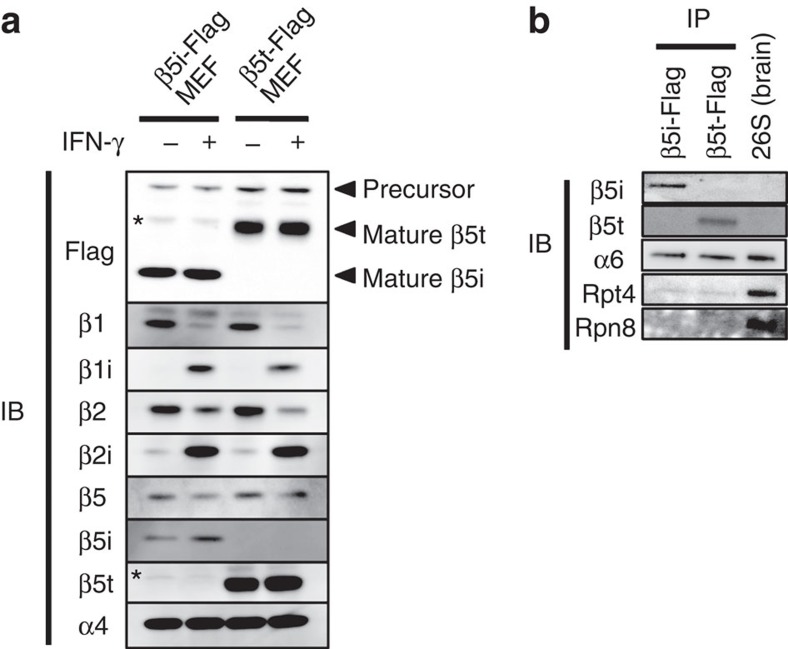
Establishment of cells predominantly expressing the tCPs. (**a**) Immunoblot of β5i-Flag or β5t-Flag MEFs cultured in the presence or absence of IFN-γ. Asterisks denote nonspecific bands. Notes that α4 is a subunit shared by all types of CPs. (**b**) Anti-Flag immunoprecipitation followed by immunoblot of CPs from β5i-Flag and β5t-Flag MEFs treated with IFN-γ. Data are representative of three independent experiments.

To understand the difference in catalytic properties between tCPs and iCPs, it is essential to obtain cells that predominantly express tCPs as CPs. However, cTECs constitute a very small population in mice (∼1 × 10^4^ cells per mouse). To obtain tCPs without contamination of iCPs, we introduced β5t or β5i with a C-terminal Flag-tag into immortalized *β5i*^−/−^ murine embryonic fibroblasts (β5t-Flag or β5i-Flag MEFs, respectively). When these cells were treated with IFN-γ, the majority of β1 and β2, which were predominantly expressed in the absence of IFN-γ, were replaced by β1i and β2i (Fig. 1a). Together with exogenously expressed β5i-Flag or β5t-Flag, either the iCP or the tCP was assembled in these cells (Fig. 1a). We purified iCPs and tCPs from these cells by anti-Flag immunoprecipitation at high salt concentration to dissociate CP activators. Immunoblotting with Rpt4 and Rpn8 revealed that the majority of the purified CPs were free of the 19S regulatory particle, in contrast to the 26S proteasome purified from the brain (Fig. 1b).

### tCP generates unique peptides *in vitro*

To compare cleavage specificities, three proteins (chicken ovalbumin (OVA), yeast enolase and bovine β-casein) were subjected to *in vitro* digestion by the purified iCPs and tCPs (Fig. 1b), followed by sequence determination of the resultant peptides using mass spectrometry. To minimize secondary cleavage caused by re-entry of degradation products, peptides were recovered at time points when ∼50% of each substrate was degraded (Supplementary Fig. 1). Peptides detected in all three independent experiments with less than a 5% false discovery rate were used for analysis. iCPs and tCPs produced a total of 143 and 245 different peptides from the substrates (Table 1 and Supplementary Tables 1–3). Of these, 97 peptides were produced by both iCPs and tCPs. Accordingly, 60% (148/245) of peptides generated by tCPs and 32% (46/143) of peptides generated by iCPs were tCP- and iCP-specific, respectively. These results indicate that the peptide repertoire generated by tCPs was quite different from that generated by iCPs. Peptides generated by both likely included peptides processed by β1i and β2i.

We next determined 69 iCP-specific and 164 tCP-specific cleavage sites according to a premise where each iCP- or tCP-specific peptide is excised from a protein just before its N terminus and after its C terminus (Table 1). Amino-acid frequencies surrounding the cleavage sites were counted, with the positions and the cleavage site defined as …P3-P2-P1-P1′-P2′-P3′…, where cleavage occurs between P1 and P1′. As a reference, amino-acid frequencies in the three substrates that would be expected to occur when they were randomly cleaved were also counted.

**Figure 2:**
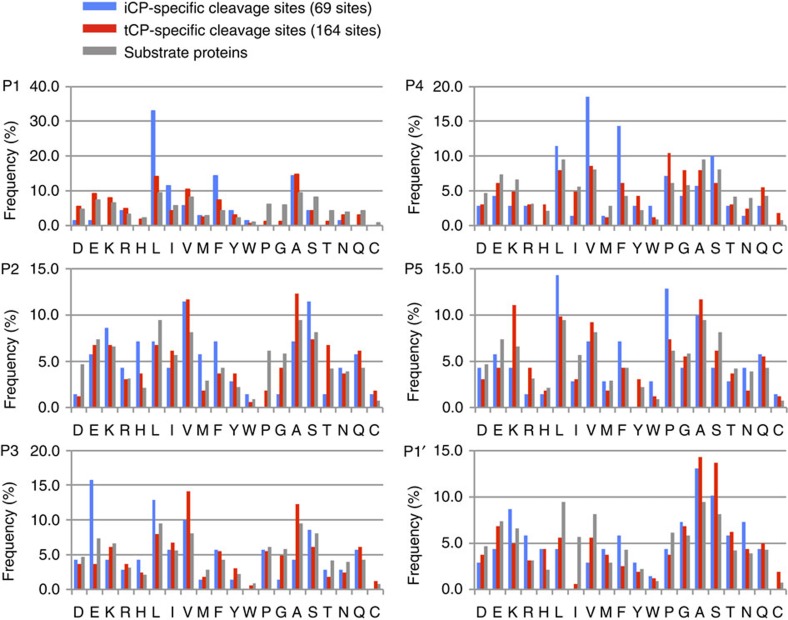
Unique amino-acid sequences of β5t-dependent peptides generated *in vitro*. Substrate proteins were digested by purified iCPs and tCPs *in vitro*. Identified cleavage sites generated specifically by the iCPs (blue) and by the tCPs (red) were compared. Amino-acid frequencies at P1–P5 and P1′ are shown. Grey bars depict amino-acid compositions of the substrates. *In vitro* digestion assay was repeated at least three times.

As expected, the P1 positions of iCP-specific peptides skewed towards hydrophobic amino acids, especially L/F (Fig. 2), consistent with previous reports^20^. In contrast, tCPs showed a much lower usage of L/F at P1, supporting the prediction that tCPs are less potent in generating peptides with hydrophobic C termini (Fig. 2 and Supplementary Table 4)^13^. Rather, the amino-acid frequencies at P1 of tCP-specific peptides were similar to amino-acid frequencies of the substrate proteins. These data suggest that tCPs do not have a specific preference for P1 and cleave a peptide bond after any amino acid, yet tCPs are capable of generating MHC-I-binding peptides since they can cleave at the C terminus of hydrophobic amino acids, which is required for binding to most alleles of MHC-I in mice^21^,^22^.

The amino acid at P1 is not the only determinant for a cleavage site by CPs. It has been suggested that proteasomal cleavage is influenced by residues flanking the cleavage site on either side^20^,^23^,^24^. Indeed, differences in amino-acid usage were found between iCPs and tCPs, especially at P3, P4 and P5 positions, where iCPs strongly preferred E at P3, V or F at P4 and P at P5, while tCPs preferred several amino acids including P at P4 and K at P5 (Fig. 2 and Supplementary Table 4). These results indicate that the protease functions of β5t and β5i influence amino-acid distributions not only at P1 but also at the N-terminal flanking residues, especially at P3, P4 and P5, and suggest that tCPs generate peptides with properties distinct from iCPs. This difference may influence amino-acid sequence of MHC-I-binding peptides.

### Altered presentation of SIINFEKL peptides by the tCP

**Figure 3:**
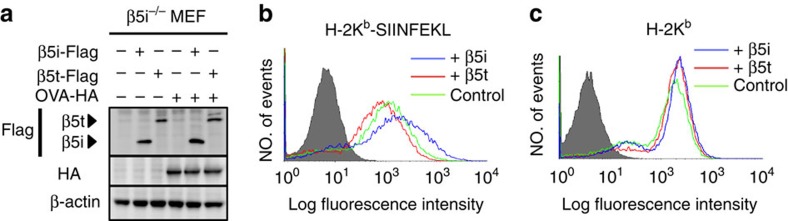
Poor presentation of OVA_257–264_ epitope by tCPs. (**a**) A plasmid encoding chicken OVA with C-terminal HA-tag was stably transfected into β5i^−/−^, β5i-Flag and β5t-Flag MEFs. Extracts of the cells treated with IFN-γ were analysed by immunoblot using the indicated antibodies. (**b**) β5i-Flag (blue), β5t-Flag (red) and β5i^−/−^ (green) MEFs with OVA transgene and IFN-γ treatment were analysed using flow cytometry for surface expression of SIINFEKL-presenting H-2K^b^ and (**c**) total H-2K^b^. The grey histograms denote negative controls without primary antibodies. Data are representative of three independent experiments.

To confirm whether such sequence preferences are true in a biologically relevant situation, we examined the presentation of an OVA-derived H-2K^b^-binding epitope, OVAp (SIINFEKL)^25^,^26^, which should be preferably processed by iCPs because this epitope has a motif preferred by iCPs rather than tCPs: L at P1, E at P3 and F at P4. We introduced OVA cDNA into β5i-Flag MEFs and β5t-Flag MEFs as well as *β5i*^−/−^ MEFs and cultured them in the presence of IFN-γ to induce iCPs and tCPs. The three MEF lines expressed nearly equivalent amounts of OVA proteins (Fig. 3a). However, the expression of the H-2K^b^-OVAp complex probed by a specific antibody 25-D1.16 was clearly reduced in β5t-Flag MEFs (MFI=3,185), compared with β5i-Flag MEFs (MFI=9,331) and even compared with *β5i*^−/−^ MEFs (MFI=4,613), which express neither iCPs nor tCPs (Fig. 3b). This difference is not simply owing to the decreased chymotrypsin-like activity of β5t, because surface expression of total H-2K^b^, which requires the association of C-terminal hydrophobic peptides, was at similar levels (Fig. 3c). These results underpin the different specificity of peptide processing found in the *in vitro* experiment (Fig. 2) and suggest that the MHC-I-binding peptides presented by tCP-expressing cells are different from those presented by iCP-expressing cells owing to the different catalytic specificities.

### Generation of canonical MHC-I peptides by the tCP

**Figure 4:**
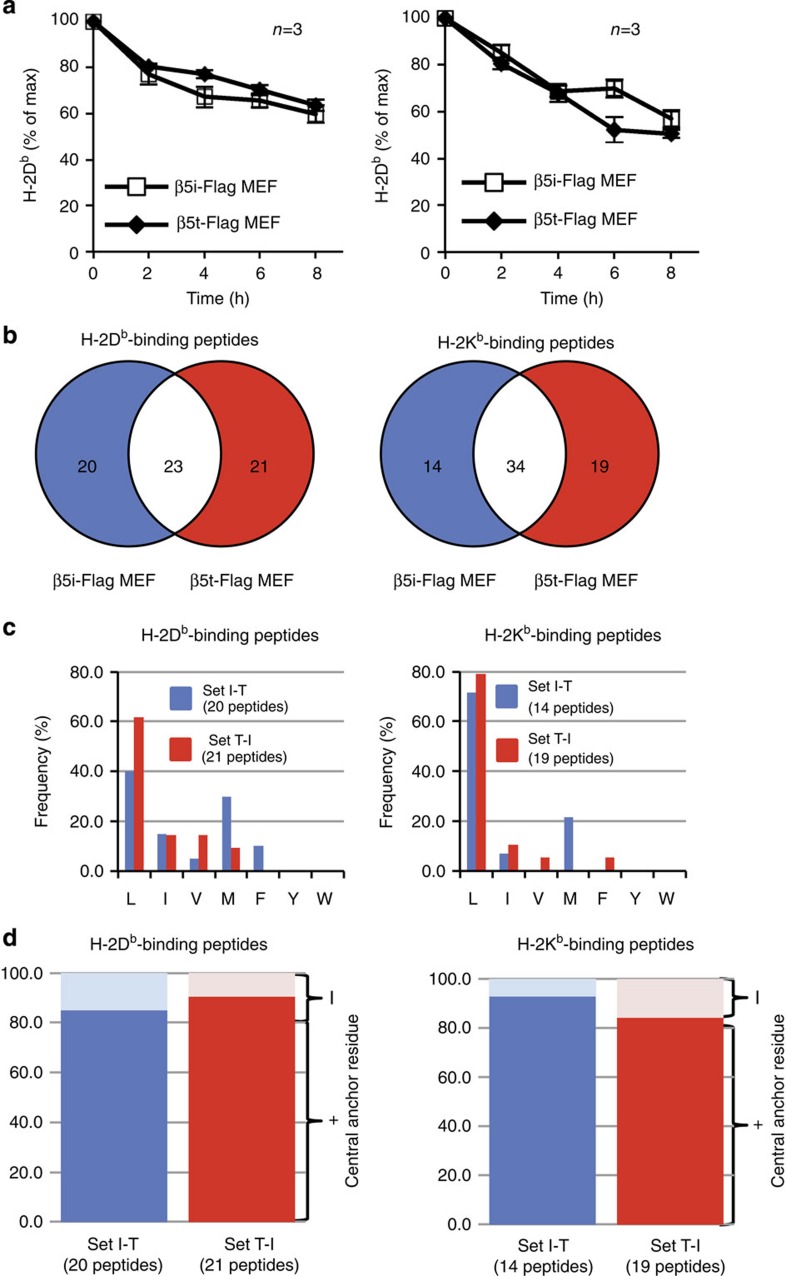
tCPs generate peptides with requirements for high-affinity MHC-I binding. (**a**) Cell surface expression of H-2D^b^ and H-2K^b^ on β5i-Flag MEFs (open square) and β5t-Flag MEFs (closed diamond) cultured with IFN-γ and brefeldin A was measured using flow cytometry. MEFs cultured without brefeldin A were used as the control of maximum (100%). Data are presented as the mean percentage±s.e.m. (*n*=3). Statistical analyses were performed by Student's *t*-test. (**b**) Summary of H-2D^b^- and H-2K^b^-binding peptides presented on β5i-Flag MEFs (blue circles; Set I) and β5t-Flag MEFs (red circles; Set T). The numbers of peptides with 8–14 lengths of residues and hydrophobic amino acids at their C termini are shown. (**c**) Frequencies of amino acids at C-terminal positions in peptides with 8–14 lengths of residues and hydrophobic amino acids at their C termini generated specifically by iCPs (blue bars; set I-T) and by tCPs (red bars; set T-I). (**d**) Frequencies of central anchors of peptides with 8–14 lengths of residues and hydrophobic amino acids at their C termini. Blue and red bars denoted as in **c**.

Given the fact that tCPs cleaved peptides differently from iCPs, we explored differences in the peptides bound to MHC-I between β5i-Flag MEFs and β5t-Flag MEFs cultured in the presence of IFN-γ by immunoprecipitation of MHC-I–peptide complexes. The use of immunoprecipitation approach may bias the data by enriching the most stable MHC-I–peptide complexes. This issue of stability was relevant because tCPs seemed less efficient than iCPs in producing hydrophobic C-terminal amino acids of typical MHC-I-binding peptides. Thus, we examined the stability of MHC-I–peptide complexes on β5i-Flag MEFs and β5t-Flag MEFs (Fig. 4a). Because dissociation of peptides from MHC-I molecules on the cell surface causes rapid internalization of MHC-I molecules, it was possible to evaluate the stability of peptide binding to MHC-I by measuring the rate of decline in H-2K^b^ and H-2D^b^ on the MEFs treated with brefeldin A, which blocks the *de novo* supply of the MHC-I–peptide complex to the cell surface^27^,^28^. We detected no significant difference in the decline rates of both H-2D^b^ and H-2K^b^ between β5i-Flag MEFs and β5t-Flag MEFs (Fig. 4a). These data suggest that the stability of the majority of cell surface-expressed MHC-I-associated peptides on tCP-expressing cells is indistinguishable from that on iCP-expressing cells.

MHC-I–peptide complexes were immunoprecipitated using anti-H-2D^b^ (28-14-8) or H-2K^b^ (Y3) monoclonal antibodies. Amino-acid sequences of peptides eluted from these complexes were determined using mass spectrometry. Peptides detected at least two times with less than a10% false discovery rate in five independent experiments were used for the following statistical analysis. The antigen-processing system usually produces MHC-I-binding peptides with lengths of 8–14 residues, and therefore we chose the peptides of 8–14 residues (Supplementary Tables 5 and 6). Consequently, 45 D^b^- and 48 K^b^-binding peptides from β5i-Flag MEFs and 49 D^b^- and 54 K^b^-binding peptides from β5t-Flag MEFs were subjected to analysis. Although tCPs cleave peptide bonds after any amino acid *in vitro* (Fig. 2), most of the identified MHC-I-binding peptides from β5t-Flag MEFs had hydrophobic C termini (L/I/V/M/F/Y/W; Supplementary Tables 5 and 6). Peptides that did not have C-terminal hydrophobic residues were excluded from further analyses as possible contaminants because it was indicated that hydrophobic amino acids at P1 serve as a primary anchor for binding to D^b^ and K^b^ and are a prerequisite for the stability of the MHC-I–peptide complex^21^,^22^.

Next, 43 D^b^- and 48 K^b^-binding peptides from β5i-Flag MEFs (Set I) and 44 D^b^- and 53 K^b^-binding peptides from β5t-Flag MEFs (Set T) that have C-terminal hydrophobic residues were subjected to further analysis. Of these, 23 D^b^- and 34 K^b^-binding peptides were generated by both MEFs, and 21 D^b^- and 19 K^b^-binding peptides were tCP-specific (Set T-I), and 20 D^b^- and 14 K^b^-binding peptides were tCP-specific (Set I-T; Fig. 4b), indicating that the MHC-I–peptide repertoire presented by tCP-expressing cells is substantially different from that presented by iCP-expressing cells.

Considering that tCPs but not iCPs enable effective positive selection in cTECs^13^,^14^,^28^, peptides produced specifically by tCPs (Set T-I) might be more potent in inducing positive selection than peptides produced specifically by iCPs (Set I-T). Therefore, clarifying differences between tCP-specific and iCP-specific peptides is the key to understanding the mechanism of positive selection.

The MHC-I-binding peptides are restricted in length and often contain the key anchor residues at particular positions. In peptides with hydrophobic P1 residues, the amino-acid usage at P1 in the tCP-specific peptides was similar to that in peptides generated by the iCP, L being the most abundant residue in both MHC-I alleles (Fig. 4c). Another main anchor (central anchor) for D^b^ and K^b^ is located, respectively, at P5 and P4 of each peptide, which are occupied by N in D^b^-binding peptides and F/Y in K^b^-binding peptides^21^,^29^. Of the peptides with a hydrophobic P1, more than 80% of the peptides generated by either iCPs or tCPs had central anchors (Fig. 4d). These results suggest that tCPs produce peptides equipped with a canonical requirement for high-affinity MHC-I-binding, yet generating a MHC-I–peptide repertoire different from that by the iCP.

### tCP produces a unique MHC-I–peptide repertoire

**Figure 5:**
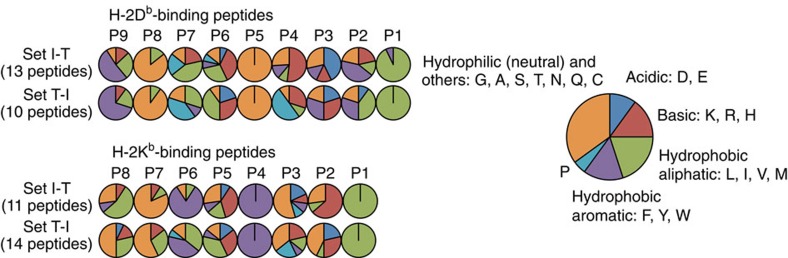
Distinct amino-acid usage of tCP-specific MHC-I peptides. Amino-acid frequencies of each position in canonical H-2D^b^-binding and H-2K^b^-binding peptides. 9-mer peptides with hydrophobic C termini and N at P5 for H-2D^b^ and 8-mer peptides with hydrophobic C termini and F or Y at P4 for H-2K^b^ were used for the analysis.

We speculated that differences between the tCP-specific and iCP-specific peptides lay in regions flanking the anchor residues even in canonical peptides. In order to deduce tCP- and iCP-specific motifs as precisely as possible, we selected peptides with the following criteria for further analysis: detected at least twice in five independent experiments, a typical peptide length (9-mer for D^b^ and 8-mer for K^b^), and contains both hydrophobic C terminus and the central anchor residue. Consequently, we focused on 13 D^b^- and 11 K^b^-binding canonical peptides from set I-T, and 10 D^b^- and 14 K^b^-binding canonical peptides from set T-I (Supplementary Tables 5 and 6). We grouped amino acids into acidic (D/E), basic (K/R/H), hydrophobic aliphatic (L/I/V/M), hydrophobic aromatic (F/Y/W), hydrophilic neutral (G/A/S/T/N/Q/C) and proline (P), and compared amino-acid frequencies at each position of the peptides. With respect to D^b^-binding peptides, we found notable differences at P3 and P4 (Fig. 5 and Supplementary Table 7). Other than the anchor residues, it is known that acidic residues frequently occupy P3 of D^b^-binding peptides^21^,^30^. Indeed, the iCP-specific peptides enriched acidic residues at this position (43%). In contrast, hydrophobic, especially aromatic, residues and basic residues were predominant at P3 of the tCP-specific peptides, while acidic residues were reduced (Fig. 5 and Supplementary Table 7).

Another remarkable difference was enrichment of P at P4, adjacent to the central anchor, in the tCP-specific peptides (50%). Regarding K^b^-binding peptides, we found enrichment of P at P3, adjacent to the central anchor, and preference for acidic residues at P2 in the tCP-specific peptides (Fig. 5 and Supplementary Table 8). These results demonstrate that the tCP generates peptides that use distinct amino acids near the central anchor residues for MHC-I binding.

### Peptides with tCP-dependent motifs exhibit low TCR affinity

**Figure 6:**
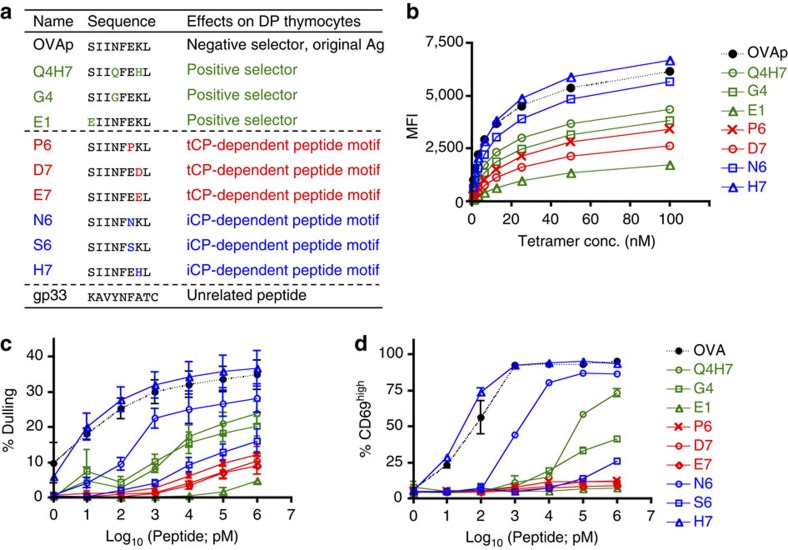
OVAp peptide variants with tCP-dependent motifs show lower affinities to OT-I TCR. (**a**) List of OVAp variants used for assays in **b**–**d** and Fig. 7a,b. (**b**) Binding of peptide/H-2K^b^ tetramers to CD4/CD8 double-positive thymocytes from *Tap1*^−/−^ *OT-I TCR-*transgenic mice. (**c**) Dulling of CD4/CD8 co-receptors in OT-I thymocytes by OVAp variants. Thymocytes from *Tap1*^−/−^ *OT-I TCR*-transgenic mice (*n*=3) were stimulated in the presence of peptides at indicated concentrations and irradiated splenocytes from B6-Ly5.1 mice. (**d**) Activation of mature OT-I cells by OVAp variants. Splenocytes from *Rag2*^−/−^ *OT-I TCR* transgenic mice (*n*=3) were cultured in the presence of peptides at indicated concentrations for 5 h. Representative data from three (**b**) and four (**c**,**d**) independent experiments.

To examine whether these peptide features play roles in thymic selection, we employed the OVA-specific TCR transgenic line OT-I, which is the best-studied model of amino-acid substitution in MHC-associated peptides for thymic selection of functionally competent MHC-I-restricted T cells. The antigenic H-2K^b^-binding OVAp (SIINFEKL), which exhibits a high-affinity binding to OT-I TCR, mediates negative selection of OT-I thymocytes, while its variant peptides Q4H7, G4 and E1, which have low affinities to OT-I TCR, promote positive selection (Fig. 6a). We then tested OVAp variant peptides with tCP-dependent motifs, that is, P at P3 (P6) and D/E at P2 (D7/E7), and variants with iCP-dependent motifs, N at P3 (N6), S at P3 (S6) and H at P2 (H7). As previously reported^31^, the positive selectors Q4H7, G4 and E1 bound to OT-I TCR at lower affinities than OVAp when analysed using MHC-tetramer-binding assay (Fig. 6b), CD4/CD8 dulling assay (Fig. 6c) and agonist activity assay (Fig. 6d). Notably, while H7 and N6 with iCP-dependent motifs exhibited high affinity to OT-I TCR like OVAp, P6 and D7 with tCP-dependent motifs and S6 with an iCP-dependent motif showed low affinities, similar to the known positive selector peptides (Fig. 6b–d).

We also performed the CD4/CD8 dulling assay and the TCR agonist assay using peptide variants and two other transgenic TCRs, P14 and F5. The peptide ligands for these TCRs are associated with H-2D^b^; therefore, P6 (P at P4) and K6 (K at P4) variants were tested as tCP- and iCP-motif peptides, respectively. We found that in both P14 and F5 TCR ligand peptides, the introduction of the iCP motif K6 to original antigenic peptides completely disrupted the dulling and agonistic activities of original peptides specifically recognized by these TCRs (Supplementary Fig. 2). On the other hand, the introduction of the tCP motif P6 sustained the weak interaction of variant peptides with P14 TCR, similarly to other positive selection-inducing peptides, but not with F5 TCR (Supplementary Fig. 2). Collectively, our findings on these tCP and iCP motif peptides on three different TCRs indicate that four out of five tCP motif peptides exhibit low affinity to TCR, whereas only one of five iCP motif peptides exhibits low affinity to TCR (Supplementary Table 9). These results indicate that tCP- and iCP-dependent motifs differently affect affinity between peptides and TCRs, and suggest that low-TCR-affinity peptides, which can efficiently induce positive selection, are preferentially produced by tCP rather than by iCP, at least in antigen-specific TCR-transgenic models, although tCP does not always produce low-affinity peptides and iCP does not always fail to produce low-affinity peptides.

### tCP-dependent motifs promote CD8^+^ T-cell-positive selection

**Figure 7:**
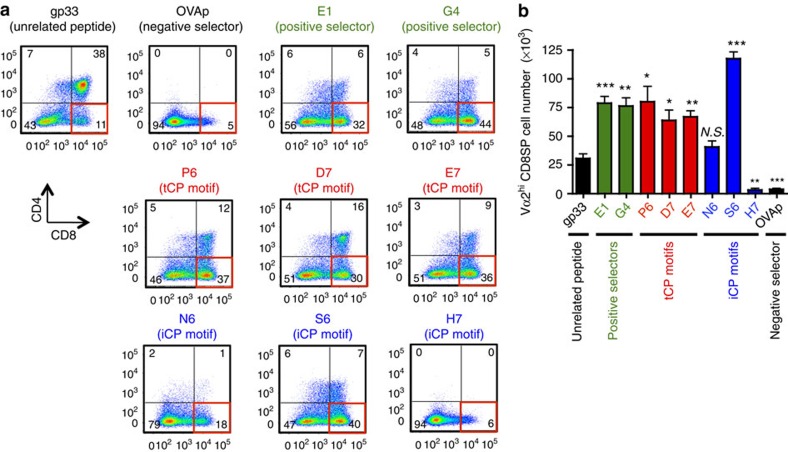
OVAp peptide variants with tCP-dependent motifs promote the positive selection of OT-I CD8^+^ T cells. (**a**) FTOC was performed using E15 thymus lobes from *Tap1*^−/−^ *OT-I TCR* transgenic mice in the presence of the indicated peptides (listed in Fig. 6a). Three days after the beginning of the culture, cells were harvested and analysed using flow cytometry. Representative CD4 versus CD8 plots from three independent experiments and (**b**) average numbers±s.e.m. (*n*=3) of Vα2^high^ CD8 single-positive (SP) thymocytes per thymus lobe are shown. Cumulative data of three to four thymus lobes per experimental group are shown in **b**. ***P<0.01, **P<0.01, *P<0.05, N.S. not significant in comparison with the cell number from the culture in the presence of gp33 peptide. Statistical analyses were performed by Student's *t*-test.

We further tested the effects of those tCP- and iCP-dependent OVAp variant peptides in fetal thymus organ culture (FTOC) of OT-I thymocytes on a background deficient for the peptide transporter TAP1 to evaluate the thymic selection dependent on the addition of exogenous peptides. We found that all three tCP-dependent motif peptides, P6, D7 and E7, induced large fractions of CD8 single-positive cells, quite similar to the profiles brought by the positive selector peptides, E1 and G4 (Fig. 7a). The total numbers of OT-I TCR^high^ CD8 single-positive thymocytes induced by P6, D7 and E7, as well as the positive selector peptides, were significantly larger than the unrelated peptide gp33 (Fig. 7b). In contrast, three iCP-dependent motif peptides behaved quite differently from each other. Addition of N6 to the FTOC induced no significant effect onto the background cellularity of TCR^high^ CD8 single-positive thymocytes in the presence of unrelated gp33 (Fig. 7b); addition of S6 induced a clear increase in the generation of TCR^high^ CD8 single-positive thymocytes, similar to other positive selection-inducing peptides (Fig. 7b); and addition of H7, as well as OVAp, to the FTOC induced a clear decrease in CD8 single-positive cells accompanied by depletion of CD4/CD8 double-positive cells, a hallmark of negative selection (Fig. 7a,b). These results indicate that tCP- and iCP-dependent motifs differently promote positive selection of CD8^+^ T cells and suggest that tCP motif peptides are enriched for low-affinity peptides, whereas iCP motif peptides exhibit highly diverse TCR affinities from undetectable affinity to high affinity.

Finally, we examined CD5 expression in polyclonal TCR-expressing thymocytes generated in the presence or absence of tCPs. It is well known that CD5 expression levels in mature single-positive thymocytes correlates with the affinity of the TCR to its peptide–MHC ligand during positive selection^32^,^33^. We found that in tCP-deficient mice, in which iCP compensates the loss of tCP in cTECs, CD5 expression by mature TCR^high^ CD8 single-positive thymocytes, but not mature TCR^high^ CD4 single-positive thymocytes, was higher than that in control mice, in which tCP is normally expressed in cTECs (Supplementary Fig. 3). These results suggest that abnormal positive selection induced in the absence of tCP, which is presumably induced by iCP-expressing cTECs, is predominantly induced by the peptide ligands that exhibit different TCR affinity from the peptides normally expressed by tCP-expressing cTECs. It is therefore suggested that tCP preferentially produces low-TCR-affinity peptides that are optimal for positive selection of a wide range of functionally useful TCR specificities.

## Discussion

In this study, we demonstrated that the tCP has a unique catalytic activity that is not shared by the iCP, and this feature resulted in presentation of a unique peptide repertoire by MHC-I of tCP-expressing cells. The primary amino-acid sequence corresponding to the substrate-binding pocket of β5t and an experiment using fluorogenic short peptides suggested that β5t has very weak chymotrypsin-like activity^13^. However, an *in vitro* protein degradation assay revealed that β5t can generate peptides with C-terminal hydrophobic amino acids (Fig. 2), and this activity was sufficient to generate peptides that can bind to MHC-I since the tCP-expressing cells present a comparable amount of MHC-I–peptide complexes to the iCP-expressing cells (Fig. 3c)^13^,^14^.

This catalytic feature of β5t enables the tCP-expressing cells and probably cTECs to present MHC-I-associated peptides with authentic anchor residues and yet with unique P2, P3 and/or P4 residues (Fig. 5). We found no difference in the decline rate of H-2D^b^ and H-2K^b^ between β5i-Flag MEFs and β5t-Flag MEFs (Fig. 4a). Indeed, it has been previously reported that the half-lives of H-2D^b^ and H-2K^b^ were similar between cTECs (predominantly expressing tCPs) and medullary thymic epithelial cells (predominantly expressing iCPs)^28^. In addition, we previously reported that β*5t*^−/−^ cTECs (predominantly expressing iCPs) and wild-type cTECs (predominantly expressing tCPs) express similar amounts of peptide-bound and peptide-empty forms of H-2L^d^ on the cell surface, with most H-2L^d^ on *β5t*^−/−^ and wild-type cTECs being associated with peptides^14^. This suggests that large fractions of peptides generated by tCP- and iCP-expressing cells have similar stability of binding to MHC-I. However, we acknowledge that it is possible that the identification of MHC-associated peptides using immunoprecipitation may cause biased analysis of only a stable fraction of MHC-I–peptide complexes.

Crystal structural analysis of MHC-I–peptide–TCR complexes has shown that the peptide aligns in a linear and extended manner, with side chains of anchor residues pointing downwards to interact with the MHC, while others are pointing upwards to contact the TCR^34^,^35^,^36^. With respect to H-2K^b^, the residues between the central and C-terminal anchors bulge out of the MHC-I cleft, exposing P2 and P3 side chains to interact with the CDR3 loop of TCR^26^,^37^. Therefore, peptides generated specifically by tCPs should exhibit interactions with TCRs that are different from those mediated by peptides generated by iCPs. These interactions may induce a signalling pathway downstream of an engaged TCR that results in survival of the immature T cell. Indeed, our data suggested that the K^b^-associated OVAp variants that carried the tCP-specific amino-acid motifs preferentially exhibited low affinity to OT-I TCR and were efficient in inducing the positive selection of OT-I T cells (Figs 6 and 7). This suggestion was not only supported in OT-I TCR experiments but also in other TCRs, including a polyclonal T-cell population (Supplementary Fig. 3). Moreover, a previous report showed that the tCP motif peptides, P6 and E7, tend to moderately weaken the agonistic activity to T cells^38^. However, since we deduced the motifs on the basis of the analysis of peptides that have all the typical characteristics of canonical peptides regarding peptide length, C termini and central anchors, the motifs may be valid only for very typical H-2D^b^- and H-2K^b^-bound peptides. Therefore, we need to be careful in generalizing the results to the whole H-2D^b^ and H-2K^b^ peptide population.

It was previously hypothesized that MHC-I–peptide complexes displayed by cTECs are different from other antigen-presenting cells^13^,^14^,^28^. However, it remained to be determined whether the difference in repertoire between peptides generated by tCPs-generated and peptides generated by iCPs or cCPs is important for inducing the positive selection of CD8^+^ T cells, or the peptides carrying tCP-dependent motifs are equipped with features advantageous for inducing optimal positive selection of CD8^+^ T cells. It has been reported that MHC-I–peptide complexes generated by iCP in tCP-deficient cTECs failed to induce the positive selection of CD8^+^ T cells, even when medullary thymic epithelial cells displayed a different peptide set generated by cCP, which supports the notion that peptides generated specifically by tCPs carry a unique feature for promoting positive selection^28^. Our results further support the specialty of peptide set on cTECs. Indeed, our results suggest that tCPs directly contribute to the positive selection of functionally competent CD8^+^ T cells by providing a unique repertoire of MHC-I-associated self-peptides that are suitably low affinity to TCR at least in the case of model protein antigens, rather than simply providing a cTEC-specific peptide repertoire that is different from peptide sets displayed by other cells.

## Methods

### Cell culture

MEFs and PlatE cells were cultured in DMEM supplemented with 10% fetal bovine serum. MEFs were obtained from β5i-deficient mice (C57BL/6), immortalized by introducing the SV40 large T antigen, and cultured as described previously^39^. Retroviruses were produced and used for infection as described previously^40^. In brief, a pMXs-IRES-Puro plasmid encoding Flag-tagged mouse β5i or β5t and a pMXs-IRES-Bsd encoding chicken OVA were introduced into PlatE cells by conventional calcium phosphate transfection. The medium was changed 24 h after transfection, and the culture supernatant containing infectious retrovirus was collected 48 h after transfection. MEFs were infected with retroviral containing media in the presence of 4 μg ml^−1^ polybrene. To generate stable cell lines, transformed MEFs were selected with 4 μg ml^−1^ puromycin and/or 12 μg ml^−1^ blasticindin. To induce iCPs and tCPs, the cells were stimulated with IFN-γ (Peprotech) at 50 units per ml for 96 h.

### Western blot analysis

Cells were washed with cold PBS and lysed in a buffer containing 25 mM Tris-HCl (pH 7.5), 1 mM dithiothreitol (DTT), 2 mM ATP, 5 mM MgCl_2_ and 0.2% NP-40. Extracted or immunoprecipitated proteins were boiled with 4 × SDS loading buffer and electrophoresed in a 12.5% polyacrylamide gel and transferred to polyvinylidene difluoride membranes. Membranes were soaked in Blocking One (nacalai tesque) and immunoreacted with the primary antibodies and secondary antibodies conjugated to horseradish peroxidase (Jackson ImmunoResearch, dilution 1:20,000). Images have been cropped for presentation. Protein bands were visualized by incubation with Western Lightning Plus-ECL (Perkin Elmer), and images were acquired using ImageQuant LAS 4000mini (GE Healthcare). Uncropped images of blots and gels are presented in Supplementary Fig. 4.

### *In vitro* digestion assay

OVA, yeast enolase and bovine β-casein (Sigma-Aldrich) were denatured, reduced and S-carboxymethylated as described previously^41^,^42^. They were dialysed against 0.1 M Tris-HCl (pH 8.0) and stored at −80 °C until use. For purification of the proteasome, β5i-Flag and β5t-Flag MEFs treated with IFN-γ were lysed in a buffer containing 25 mM Tris-HCl (pH 7.5), 1 mM DTT, 2 mM ATP, 5 mM MgCl_2_ and 0.2% NP-40. Equal amounts of each protein were subjected to immunoprecipitation with M2 agarose (Sigma), followed by elution with the FLAG peptide. OVA (7 μg), yeast enolase (20 μg) and bovine β-casein (10 μg) were mixed with the purified proteasomes (1 μg) and incubated at 37 °C. Degradation time points of OVA, yeast enolase and bovine β-casein by iCPs were 12 h, and 120 and 95 min, respectively. Degradation time points of OVA, yeast enolase and bovine β-casein by tCPs were 12 h, and 50 and 95 min, respectively.

### Analysis of peptides generated *in vitro*

Peptides detected in all three independent experiments with less than a 5% false discovery rate were used for analysis, followed by extracting iCP- and tCP-specific peptides. iCP- and tCP-specific cleavage sites were determined, according to a premise where each iCP- or tCP-specific peptide is excised from a substrate protein just before its N terminus and after its C terminus. Amino-acid frequencies surrounding the cleavage sites were counted, with the positions and the cleavage site defined as …P3-P2-P1-P1′-P2′-P3′…, where cleavage occurs between P1 and P1′. Amino-acid frequencies in the three substrates that would be expected to occur when they were randomly cleaved were also counted and used as a reference.

### Purification of MHC-I-binding peptides

β5i-Flag and β5t-Flag MEFs (1 × 10^9^) treated with IFN-γ were lysed in a buffer containing 50 mM Tris-HCl (pH 8.0), 300 mM NaCl and 0.5% NP-40. The lysates were subjected to immunoprecipitation using monoclonal antibodies against H-2D^b^ (28-14-8) and H-2K^b^ (Y3) to isolate MHC-I–peptide complexes. Peptides bound to MHC-I were eluted by treating the immunoprecipitates with 0.1% trifluoroacetic acid.

### Analysis of MHC-I-binding peptides

Peptides with lengths of 8–14 residues that were detected at least twice with less than a 10% false discovery rate in five independent experiments were extracted. Only peptides that have C-terminal hydrophobic residues were used for analysis, followed by extracting iCP- and tCP-specific peptides. These peptides were used for analysis of types of hydrophobic amino acids at the C terminus and analysis of central anchor frequencies. Only peptides with the canonical length (9-mer for D^b^ and 8-mer for K^b^) and the canonical central and C-terminal anchors in peptides were used for analysis of amino-acid usage in iCP- or tCP-specific peptides.

### Peptide sequence

*In vitro* digested samples and peptides eluted from MHC-I were loaded on a Microcon YM-10 (Millipore) and centrifuged. The peptides in the flowthrough fraction were purified with Zip Tip C18 (Millipore) and fractionated with reverse-phase HPLC using a DiNa direct Nano-flow LC system (KYA Tech). Each fraction was mixed with α-cyano-4-hydroxycinnamic acid (Bruker Daltonics), spotted on a MALDI target plate and analysed using ABI5800 MALDI-TOF/TOF mass spectrometry (Applied Biosystems). Peptide sequences were determined using the ProteinPilot software (Applied Biosystems) and Swiss-Prot database.

### Antibodies

Antibodies against β-actin (MAB1501R, dilution 1:500), Flag-tag (M2, dilution 1:1,000) and HA-tag (#561, dilution 1:500) were purchased from Chemicon, Sigma and MBL (Japan), respectively. The other antibodies against the proteasome subunits used in this study were described previously^13^,^43^ and used at a 1:1,000 dilution. Hybridoma cells (28-14-8 and Y3) were purchased from ATCC. Antibodies were purified from culture supernatants using protein G-Sepharose (GE Healthcase) and conjugated to NHS-activated Sepharose 4 Fast Flow (GE Healthcare), according to the manufacturer's protocol.

### Flow cytometry

Suspensions of MEFs were washed with cold PBS and stained in cold PBS containing 1% fetal calf serum, 2 mM EDTA and 0.02% NaN_3_ by 1-h incubation with antibodies following blocking of Fcγ receptors with CD16/CD32 antibodies (2.4G2, ascites at dilution 1:2,000). Cell fluorescence was analysed using FACS-Aria II (BD Biosciences). For analysis of lymphocytes, cell suspensions were incubated with fluorochrome-labelled antibodies for 30 min on ice following the Fc block. Data were acquired on FACSVerse flow cytometers (BD Biosciences) and analysed using the FlowJo software (TreeStar).

### Presentation of SIINFEKL peptides

MEFs were trypsinized, followed by blocking Fc receptors with anti-CD16/CD32 antibodies (2.4G2; ascites at dilution 1:2,000). Cells were labelled with antibodies against H-2K^b^ (AF6-88.5; BD Pharmingen, dilution 1:300) or 25-D1.16 antibody (eBioscience, dilution 1:300), followed by staining with Alexa488-labelled anti-mouse IgG (Jackson ImmunoResearch, dilution 1:300). Cells were analysed using FACS-Aria II.

### Decline rate of MHC-I–peptide complexes

MEFs treated with 50 units per ml IFN-γ for 114 h were treated with 5 μg ml^−1^ brefeldin A for the last 2, 4, 6 or 8 h before harvesting the cells. Cells were then trypsinized and stained with phycoerythrin-conjugated anti-H-2K^b^ (AF6-88.5; BD Pharmingen) or phycoerythrin-conjugated anti-H-2D^b^ (28-14-8; BECKMAN COULTER) antibodies, following blocking Fc receptors by anti-CD16/CD32 (2.4G2). MFI was quantified by FACS-Aria II. MEFs cultured without brefeldin A were used as the control of maximum (100%).

### Tetramer-binding assay

CD4/CD8 double-positive thymocytes from *Tap1*^−/−^ *OT-I TCR* transgenic mice (6- to 8-week-old females) were stained with indicated peptide-K^b^ tetramers (MBL) in the presence of 100 μM cytochalasin D (Sigma-Aldrich) at 37 °C for 90 min (refs 31, 44) and were analysed using flow cytometry.

### FTOC

FTOC was essentially performed as described previously^45^. Embryonic mice on day 15 of gestation were obtained from *Tap1*^−/−^ *OT-I TCR* transgenic mice. Thymus lobes were cultured on Isopore membrane filters (Millipore) placed on Gelfoam sponge (Pfizer) floated in medium. Peptides were added at a concentration of 20 μM. After 3 days in the culture, thymocytes were stained with antibodies specific for CD4 (GK1.5), CD8 (53-6.7) and Vα2 (B20.1) and analysed using flow cytometry; staining antibodies were obtained from Biolegend and were used at 10 μg ml^−1^.

### Dulling assay

Dulling assay was performed as described^45^,^46^ with minor modifications. Thymocytes from *Tap1*^−/−^ *OT-I TCR* transgenic mice were stimulated in the presence of peptides and irradiated splenocytes from B6-Ly5.1 mice (CD45.1^+^CD45.2^−^). Female mice at 6–8 weeks of age were used for experiments. After 18 h, thymocytes were stained with antibodies specific for CD4 (GK1.5), CD8 (53-6.7), CD45.1 (A20) and CD45.2 (104) and analysed using flow cytometry (antibodies were obtained from Biolegend and used at 10 μg ml^−1^). Specific dulling of CD4 and CD8 expression induced by peptide stimulation was calculated as previously described^46^.

### T-cell stimulation

Spleen cells from *Rag1*^−/−^ *OT-I TCR* transgenic mice (8-week-old females) were cultured in the presence of peptides for 5 h. CD69 expression on CD8^+^CD44^lo^ cells was analysed using flow cytometry using anti-CD69 antibodies (H1.2F3; BD Pharmingen, 10 μg ml^−1^).

### Mice

*Tap1*^−*/*−^ (ref. 47), *OT-I TCR*-transgenic^48^ and B6.SJL-*Ptprca* (B6-Ly5.1)^49^ mice were previously described. Mice were bred and maintained at our facility under specific pathogen-free conditions. *Rag1*^−/−^ *OT-I-TCR*-transgenic mice were purchased from Japan SLC and Taconic.

All animal experiments were performed after obtaining approval from the Institutional Animal Care Committee of Graduate School of Pharmaceutical Sciences, the University of Tokyo (approval number M25-19) and the Animal Experimentation Committee of the University of Tokushima (approval number 13116).

### Statistics

Statistical analyses were performed using a two-tailed Student's *t*-test after the evaluation of variance.

## Additional information

**How to cite this article:** Sasaki, K. *et al.* Thymoproteasomes produce unique peptide motifs for positive selection of CD8^+^ T cells. *Nat. Commun.* 6:7484 doi: 10.1038/ncomms8484 (2015).

## Supplementary Material

Supplementary Information Supplementary Figures 1-4 and Supplementary Tables 1-9

## Figures and Tables

**Table 1:** Summary of the number of peptides detected and analysed cleavage sites in *in vitro* digestion analysis.

	**CP**	**OVA**	**Yeast enolase**	**Bovine β-casein**	**Total**
Detected peptides	iCP	44	46	53	143
	tCP	117	64	64	245
	Produced by both	32	23	42	97
Analysed cleavage sites	iCP	22	32	15	69
	tCP	77	59	28	164

CP, core particle; iCP, immunoproteasome; OVA, ovalbumin; tCP, thymoproteasome.
