# Supplementary material for: Thymoproteasomes produce unique peptide motifs for positive selection of CD8+ T cells
Source: Nat Commun. 2015 Jun 23;6:7484. doi: 10.1038/ncomms8484 (PMC4557289; doi:10.1038/ncomms8484)
Supplement: Supplementary Information — Supplementary Figures 1-4 and Supplementary Tables 1-9 [file ncomms8484-s1.pdf]

Supplementary Figures

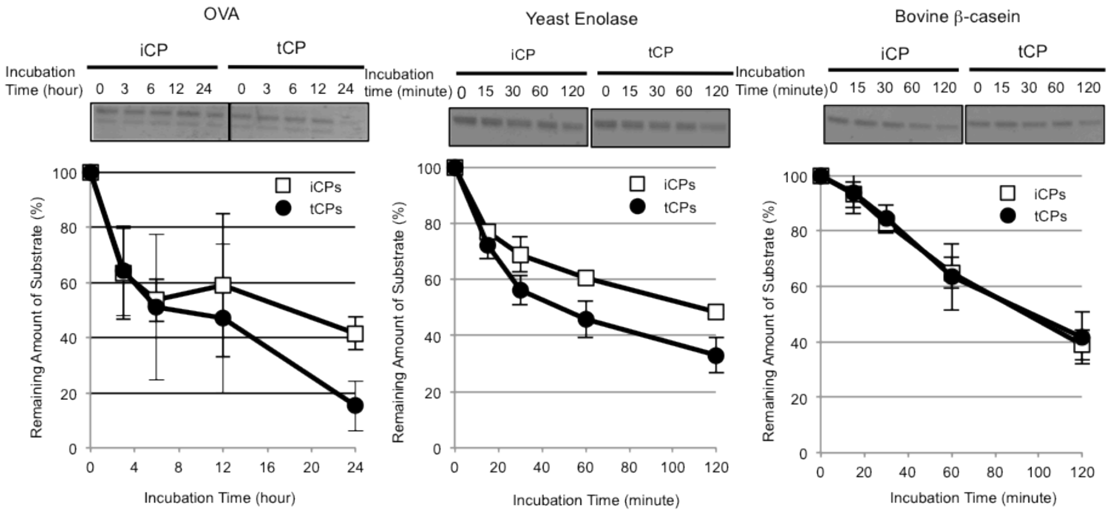

Supplementary Figure 1. Degradation rates of each of the substrates by iCPs and tCPs.

Each substrate was mixed with purified iCPs and tCPs, and incubated at 37ºC for the indicated times. The mixture was electrophoresed in a 12.5% polyacrylamide gel and stained with CBB. Bands were quantified and calculated as a percentage of the intensity of the band at time zero for each substrate. Substrates mixed with iCPs (open squares); substrates mixed with tCPs (black circles). Error bars in all panels represent mean ± SEM from three independent experiments.

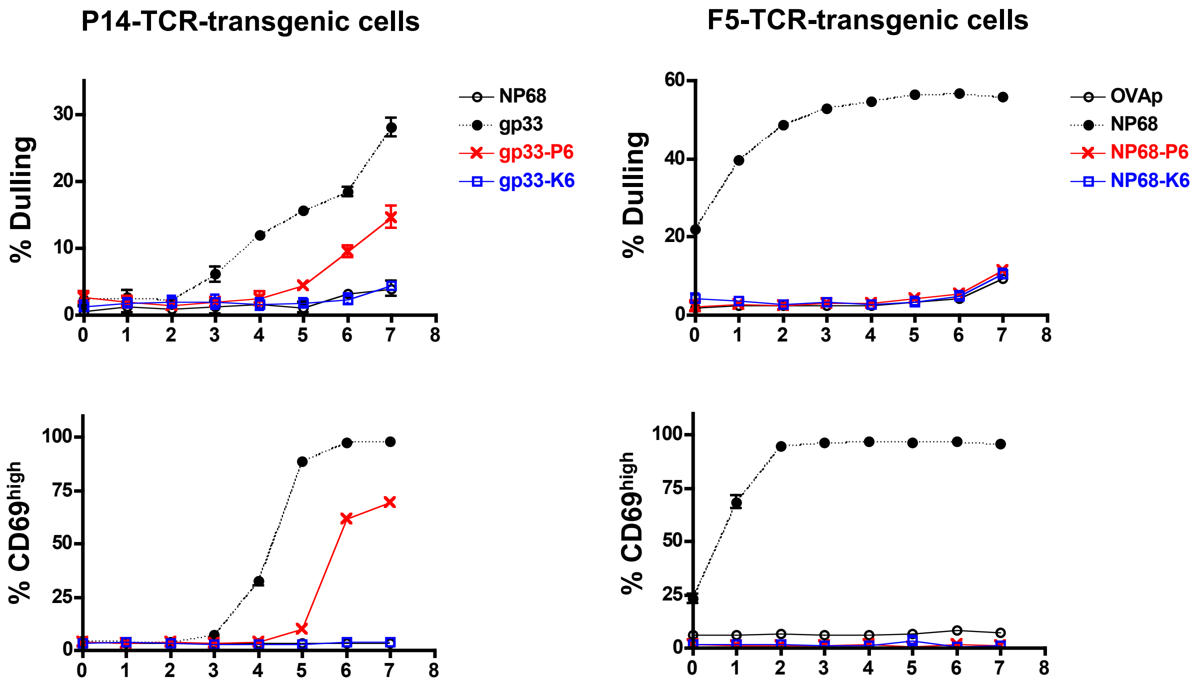

Supplementary Figure 2. Effects of tCP- and iCP-dependent motif peptides on H-2D^b-restricted TCR-expressing T cells. Lymphocytic choriomeningitis virus gp33 peptide and influenza virus NP68 peptide, which are specifically recognized by P14- and F5-TCR, respectively, were altered to contain tCP (P6)- or iCP (K6)-dependent motifs. Thymuses and spleens were obtained from P14- and F5-TCR transgenic mice, and indicated peptides were tested for co-receptor dulling in CD4$^+$CD8$^+$ thymocytes and CD69 upregulation in CD44^{lo}CD8$^+$ spleen T cells. Plotted are averages ± SEM (n = 3).

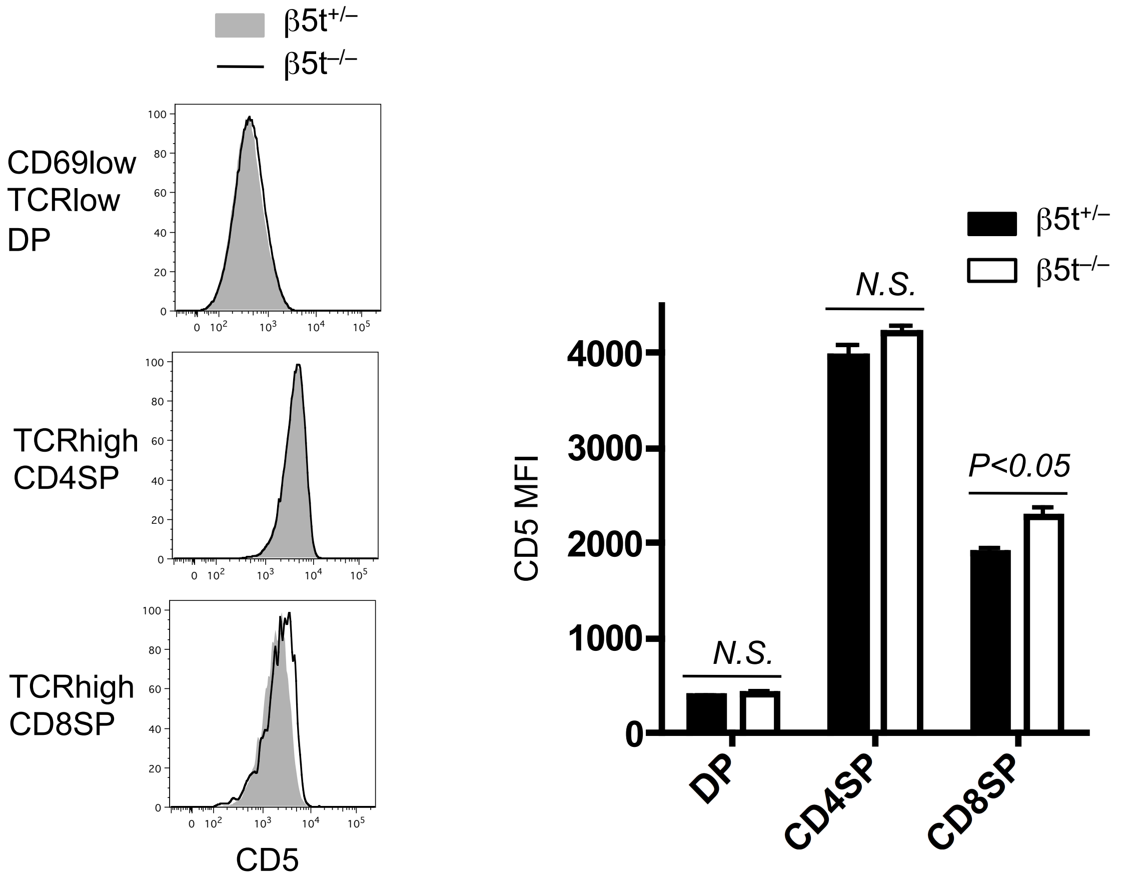

Supplementary Figure 3. CD5 expression is altered in CD8 single-positive thymocytes selected in the absence of tCPs. Thymocytes from *β5t*$^{-/-}$ and control mice were analyzed for cell-surface CD5 expression by flow cytometry. Representative histograms (left) and mean fluorescence intensity ± SEM (n = 3) in indicated populations are shown. CD4/CD8 double positive, DP; singe positive, SP. Statistical analyses were performed by Student's *t*-test.

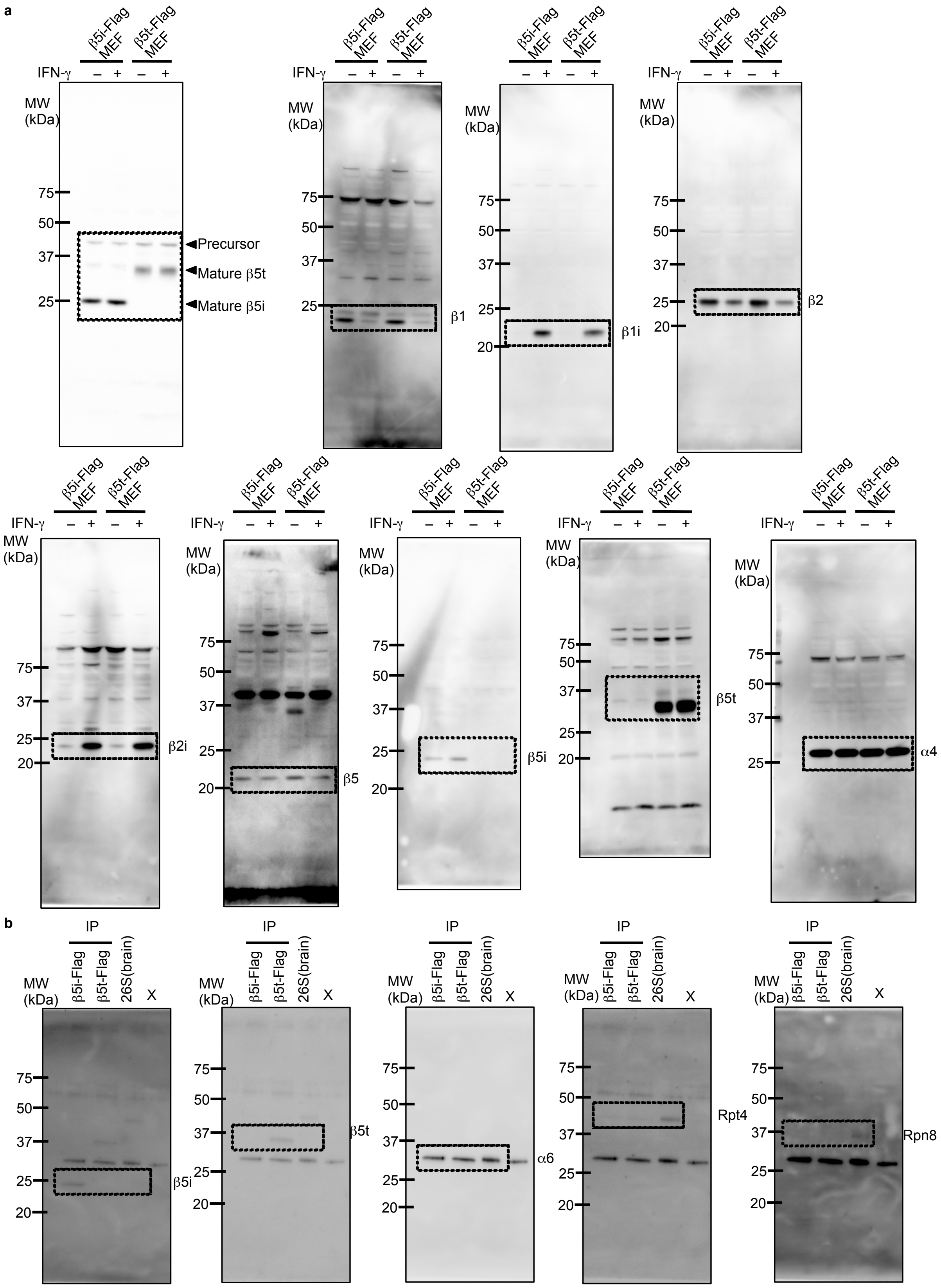

Supplementary Figure 4. Full scan of blots and gels (continued).

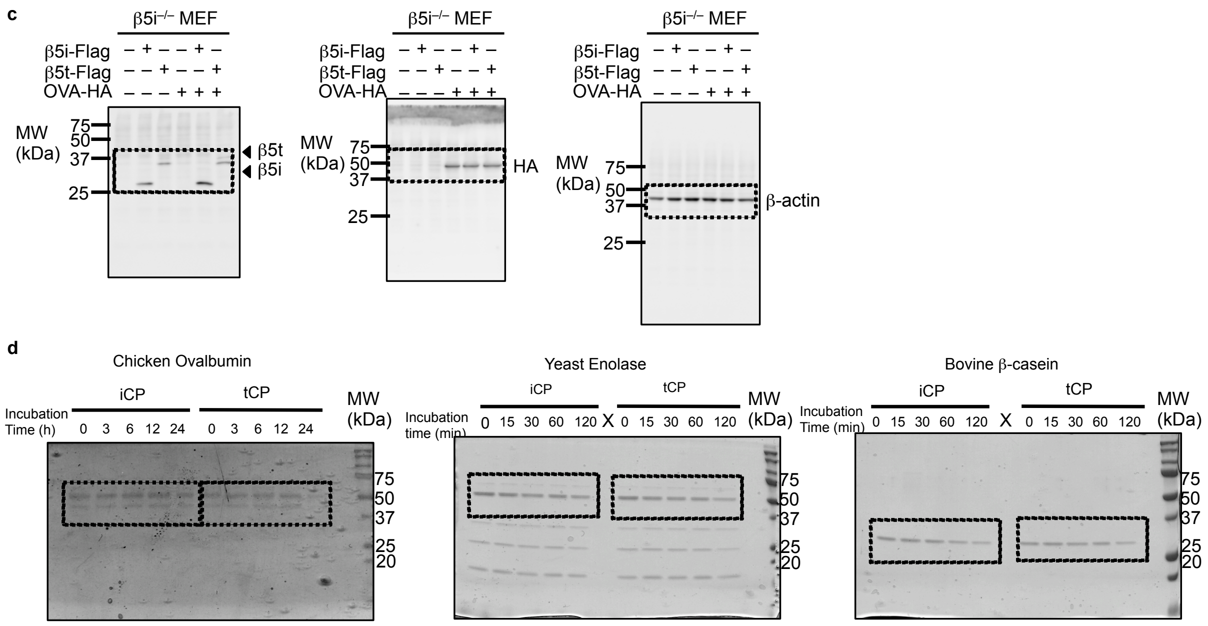

Supplementary Figure 4. Full scan of blots and gels. Uncropped images of Western blots presented in: (**a**) Fig. 1a; (**b**) Fig. 1b; (**c**) Fig.3a; (**d**) Supplementary Fig. 1. Lanes X are not shown in the corresponding Figures.

Supplementary Table 1. List of all peptides generated by iCPs or tCPs from OVA

Specifically generated by iCPs	Specifically generated by tCPs			Generated by both CPs
AGVDAASVSEEF	AAHAEINEAGREVVG	GLFRVA	QESKPVQMMYQI	AASVSEEFRADHPFL
EAGVDAASVSEEF	AAHAEINEAGREVVGSA	GVDAASVSEEFRAD	RADHPFLFCIKH	AGVDAASVSEEFRADHP
ESIINFEKL	AASVSEEFRAD	HAEINEAGREVVG	RYPILPEYL	AHAEINEAGREVVG
FKDEDTQAMPFRV	AASVSEEFRADHP	HHANENIFY	SEEFRADHPF	AHAEINEAGREVVGSA
GAKDSTRTQINKVVR	AASVSEEFRADHPF	HHANENIFYCPIAI	SEEFRADHPFLF	ASVSEEFRADHP
KELKVHHA	AASVSEEFRADHPFLFCIKH	HIATNAVLFFGRCVSP	SEEFRADHPFLFCI	ASVSEEFRADHPF
KHIATNAVL	AEERYPILPEY	HPFLF	SEEFRADHPFLFCIKH	ASVSEEFRADHPFL
KHIATNAVLFF	AEERYPILPEYL	HPFLFCI	SGISSAESLKISQAVHA	ATNAVLFFGR
MGITDVF	AFKDEDTQAMPFRV	HPFLFCIKH	SVSEEFRADHP	ATNAVLFFGRCV
NKVVRFDKL	AGVDAASVSEEFRAD	HPFLFCIKHIATN	SVSEEFRADHPFL	ATNAVLFFGRCVSP
QCVKELY	ASVSEEFRAD	HPFLFCIKHIATNA	TEQESKPVQMMY	AVLFFGR
SLASRL	ASVSEEFRADHPFLFCIKH	IATNAVLFF	TNAVLFFGR	AVLFFGRCVSP
	AVLFFGRCV	IATNAVLFFGR	TNAVLFFGRCV	EDTQAMPFRV
	DAASVSEEFRAD	IATNAVLFFGRCVSP	TNAVLFFGRCVSP	EERYPILPEYL
	DAASVSEEFRADHPFL	KAFKDEDTQAMPFRV	TQINKVVRF	FFGRCVSP
	EAGVDAASVSEEFRA	KAFKDEDTQAMPFRVTE	VHAAHAEINE	FGRCVSP
	EAGVDAASVSEEFRAD	KDEDTQAMPFRV	VHAAHAEINEAGREVVG	KHIATNAVLF
	EAGVDAASVSEEFRADHP	KDSTRTQINKVVR	VSEEFRADHPFL	KHIATNAVLFFGR
	EDTQAMPFRVTE	KELYRGGLEPINFQTA	YAEERYPILPEY	KHIATNAVLFFGRC
	EERYPIL	KHIATNAV	YLGAKDSTRTQINK	LFFGRCVSP
	EERYPILPEYLQCV	KHIATNAVLFFGRCV	YLGAKDSTRTQINKV	NAVLFFGRCVSP
	EFRADHPFL	LFFGR		RADHPFL
	ERYPILPEYL	LFFGRCV		RADHPFLFCI
	FCIKH	LYRGGLEPINF		SEEFRADHPFL
	FCIKHI	MPFRV		SIINFEKL
	FCIKHIATNA	MPFRVTE		TEQESKPVQMMYQI
	FDKLPGFGD	MYQIGLFRVA		TKPNDVYSF
	FFGRCV	NAVLFFGR		VLFFGR
	FLFCIKH	NENIFYCPIAI		VLFFGRCV
	FLFCIKHIATN	NQITKPNDVYSF		VLFFGRCVSP
	GAKDSTRTQINKV	QCVKELYRGGLEPINF		YAEERYPIL
	GAKDSTRTQINKVVRF	QESKPVQMMY		YRGGLEPINF

Supplementary Table 2. List of all peptides generated by iCPs or tCPs from yeast enolase

Specifically generated by iCPs	Specifically generated by tCPs		Generated by both CPs
AAEKNVPLYKHL	ADLSKSKTSPYVLPVPFL	NVNDVIAPAFVKA	ADLSKSKTSPYVLPVPFLNVL
AASRAAAAEKNVPLYKHL	ADLSKSKTSPYVLPVPFLN	PYVLPVPFLN	AEALRIGSEVYHNL
AIEKKAADALLLKVNQI	ADLYHSLMKRYPIV	PYVLPVPFLNVLNGGSHAGGAL	AGENFHHGDKL
AQDSFAAGWGVMV	ADLYHSLMKRYPIVSI	QDSFAAGWGVMV	ALLLKVNQIGTL
ATAIEKKAADALLLKVNQI	AEALRIGSEVYHNLK	QLLRIEEELGDN	EEELGDNAVFAGENFHHGDKL
DWEAWSHF	AGGALALQEFMIAPTGAK	QLLRIEEELGDNAVF	EELGDNAVFAGENFHHGDKL
DWEAWSHFF	ALDLIVDAIKAAGHDGKIKI	QLLRIEEELGDNAVFAGE	FMIAPTGAKTF
EDDWEAWSHFF	ALLLKVNQIGTLSE	RIEEELGDNAVFAGENFHHGDKL	GDNAVFAGENFHHGDKL
HRSGETEDTFIADLVVGL	AVSKVYAR	RSVYDSRGNPTVEV	GENFHHGDKL
KAAQDSFAAGWGVMV	AVSKVYARSV	SFAAGWGVMVSHR	NAVFAGENFHHGD
KLNQLLRIEEEL	AVSKVYARSVY	SKTSPYVLPVPFLN	NAVFAGENFHHGDKL
LNGGSHAGGALALQEF	AVSKVYARSVYD	SVYDSRGNPTVEVELTTEKGVFR	NGGSHAGGALALQEFMIAPTGAKTF
NGGSHAGGALALQEF	DSFAAG	TAEEALDLIVDAIKAA	NQLLRIEEEL
NGGSHAGGALALQEFMI	EFMIAPTGAKTF	TEKGVFRSIVPSGASTGVHEAL	PYVLPVPFL
NIDVKDQKAVDDFLIS	ELGDNAVFAGENFHHGDKL	TGAPARSERLAKLN	PYVLPVPFLNVL
NIDVKDQKAVDDFLISL	GIQIVADDLTVTNPKRIA	TGQIKTGAPARSERLAKLN	QEFMIAPTGAKTF
RIGSEVYHNL	GNPTVEVELTTEKGVFR	VSHRSGETEDTFIADLVVGL	QEFMIAPTGAKTFAEALRI
RTGQIKTGAPARSERLAKLN	GVLHAVKNVNDVIAPAFVKA	VSHRSGETEDTFIADLVVGLR	QLLRIEEELGD
SGETEDTFI	HHGDKL		QLLRIEEELGDNAVFAGENFHHGDKL
SKSKTSPYVLPVPFL	HLADLSKSKTSPYVLPVPFLN		RIEEELGDNAVF
SKSKTSPYVLPVPFLNV	HRSGETEDTFIADLVVGLR		SFAAGWGVMV
SKSKTSPYVLPVPFLNVL	KRYGASAGNVGDEGGVAPNIQ		SGETEDTFIADLVVGLR
VKNVNDVIAPAFVKA	NVNDVIAPAFVK		TAEEALDLIVDAIK

Supplementary Table 3. List of all peptides generated by iCPs or tCPs from bovine β-casein

Specifically generated by iCPs	Specifically generated by tCPs		Generated by both CPs
GVSKVKEAMAPKQKEMPFPKY	AMAPKQKEMPFPKY	PVPQKAVPYPQRD	DRQPYPVAKQPVPLVKSQS
GVSKVKEAMAPKQKEMPFPKYPVEPFTESQSL	AMAPKQKEMPFPKYPVE	PVVVPPFLQPEVM	EPVLGPVRGPFPIIV
GVSKVKEAMAPKQKEMPFPKYPVEPFTESQSLTL	AMAPKQKEMPFPKYPVEPFT	PVVVPPFLQPEVMGVSKVKE	FLLYQEPVLGPVRGPFPIIV
HLPLPLLQSW	DELQDKIHPFAQTQ	PYPQRDMPIQA	KIHPFAQTQSLVYPFPGPIPNSL
HQPHQPLPPTVMFPPQSVLSL	GPVRGPFPI	QDKIHPFAQTQ	KVLPVPQKAVPYPQRDMPIQA
MHQPHQPLPPTVMFPPQSVLSL	GPVRGPFPII	QSKVLPVPQKAVPYPQRD	LLYQEPVLGPVRGPFPIIV
PPQSVLSLSQSKVLPVPQKAVPYPQRDMPIQAF	GPVRGPFPIIV	RELEELNVPGEIVE	MAPKQKEMPFPKY
QEPVLGPVRGPFPII	KIHPFAQTQ	SKVLPVPQKAVPYPQRD	PVLGPVRGPFPII
SLSQSKVLPVPQKAVPYPQRDMPIQAFLL	KIHPFAQTQSLVYPFPGPIPN	SLSQSKVLPVPQKAVPYPQRD	PVLGPVRGPFPIIV
WMHQPHQPLPPTVMF	KVLPVPQKAVPYPQRD	SLSQSKVLPVPQKAVPYPQRDMPIQA	PVPQKAVPYPQRDMPIQA
YQEPVLGPVRGPFPIIV	KVLPVPQKAVPYPQRDMPIQAF	SLSQSKVLPVPQKAVPYPQRDMPIQAF	PVRGPFPIIV
	LGPVRGPFPIIV	SLVYPFPGPIPN	QEPVLGPVRGPFPIIV
	LPVPQKAVPYPQRD	SQSKVLPVPQKAVPYPQRD	RELEELNVPGE
	LSQSKVLPVPQKAVPYPQRD	SQSKVLPVPQKAVPYPQRDM	RELEELNVPGEIVESL
	LYQEPVLGPVRGPFPIIV	SQSKVLPVPQKAVPYPQRDMPIQ	SLSQSKVLPVPQKAVPYPQRDMPIQAFL
	MHQPHQPLPPTVMFPPQSVL	SQSKVLPVPQKAVPYPQRDMPIQA	SQSKVLPVPQKAVPYPQRDMPIQAFL
	MPIQAFLLYQEPVL	SQSKVLPVPQKAVPYPQRDMPIQAF	SQSKVLPVPQKAVPYPQRDMPIQAFLL
	PFPKYPVEPFT	TDVENLHLPLPLLQ	WMHQPHQPLPPTVMFPPQSVL
	PFPKYPVEPFTESQSL	TDVENLHLPLPLLQS	YQEPVLGPVRGPFPI
	PPFLQPEVM	VPYPQRD	YQEPVLGPVRGPFPII
	PPFLQPEVMGVSKVKE	VPYPQRDMPIQA	
	PPFLQPEVMGVSKVKEA	VPYPQRDMPIQAF	

Supplementary Table 4. Frequencies of amino acids at the N- and C-terminal positions flanking cleavage sites produced specifically by iCPs and tCPs *in vitro*

	% in substrates	iCP-specific cleavage site										tCP-specific cleavage site									
		P5	P4	P3	P2	P1	P1'	P2'	P3'	P4'	P5'	P5	P4	P3	P2	P1	P1'	P2'	P3'	P4'	P5'
D	4.7	4.3	2.9	4.3	1.4	1.4	2.9	10.3	7.4	4.4	7.4	3.1	3.1	3.7	1.2	5.5	3.7	7.5	3.8	8.2	4.4
E	7.4	5.7	4.3	15.7	5.7	1.4	4.3	7.4	10.3	5.9	13.2	4.3	6.1	3.7	6.7	9.1	6.8	6.3	8.8	8.8	7.6
K	6.6	4.3	2.9	4.3	8.6	0.0	8.7	7.4	2.9	10.3	5.9	11.0	4.9	6.1	6.7	7.9	5.0	1.3	4.4	5.7	10.1
R	3.2	1.4	2.9	2.9	4.3	4.3	5.8	2.9	1.5	4.4	4.4	4.3	3.1	3.7	3.1	4.9	3.1	3.8	5.0	5.7	5.7
H	2.1	1.4	0.0	4.3	7.1	0.0	4.3	4.4	1.5	2.9	2.9	1.8	3.1	2.5	3.7	1.8	4.3	5.6	3.1	3.1	3.2
L	9.5	14.3	11.4	12.9	7.1	32.9	4.3	10.3	10.3	4.4	2.9	9.8	8.0	8.0	6.7	14.0	5.6	12.5	6.9	6.9	4.4
I	5.6	2.9	1.4	5.7	4.3	11.4	0.0	5.9	4.4	4.4	4.4	3.1	4.9	6.7	6.1	4.3	0.6	6.9	5.7	5.7	5.1
V	8.1	7.1	18.6	10.0	11.4	5.7	2.9	4.4	5.9	10.3	11.8	9.2	8.6	14.1	11.7	10.4	5.6	9.4	8.2	5.7	8.2
M	2.9	2.9	1.4	1.4	5.7	2.9	4.3	1.5	0.0	0.0	0.0	1.8	1.2	1.8	1.8	2.4	3.7	2.5	1.3	0.6	0.0
F	4.3	7.1	14.3	5.7	7.1	14.3	5.8	1.5	1.5	0.0	4.4	4.3	6.1	5.5	3.7	7.3	2.5	6.3	6.9	5.0	5.7
Y	2.2	0.0	2.9	1.4	2.9	4.3	2.9	0.0	1.5	0.0	0.0	3.1	4.3	3.1	3.7	3.0	1.9	3.1	4.4	3.1	0.6
W	0.9	2.9	2.9	0.0	1.4	1.4	1.4	1.5	1.5	1.5	1.5	1.2	1.2	0.6	0.6	0.6	1.2	0.6	0.0	0.0	0.0
P	6.1	12.9	7.1	5.7	0.0	0.0	4.3	2.9	4.4	5.9	5.9	7.4	10.4	5.5	1.8	1.2	3.7	5.6	5.0	6.9	7.6
G	5.8	4.3	4.3	1.4	1.4	0.0	7.2	10.3	11.8	7.4	4.4	5.5	8.0	4.9	4.3	1.2	6.8	6.3	5.7	4.4	10.1
A	9.5	10.0	5.7	4.3	7.1	14.3	13.0	10.3	7.4	8.8	5.9	11.7	8.0	12.3	12.3	14.6	14.3	9.4	10.1	10.7	10.8
S	8.1	4.3	10.0	8.6	11.4	4.3	10.1	4.4	10.3	11.8	8.8	6.1	6.1	6.1	7.4	4.3	13.7	5.6	6.9	5.7	6.3
T	4.2	2.9	2.9	2.9	1.4	0.0	5.8	5.9	5.9	7.4	4.4	3.7	3.1	1.8	6.7	1.2	6.2	2.5	3.8	3.8	1.3
N	3.9	4.3	1.4	2.9	4.3	1.4	7.2	1.5	5.9	1.5	8.8	1.8	2.5	2.5	3.7	3.0	4.3	1.9	2.5	3.8	3.8
Q	4.3	5.7	2.9	5.7	5.7	0.0	4.3	5.9	4.4	8.8	1.5	5.5	5.5	6.1	6.1	3.0	5.0	1.9	5.7	5.0	4.4
C	0.8	1.4	0.0	0.0	1.4	0.0	0.0	1.5	1.5	0.0	1.5	1.2	1.8	1.2	1.8	0.0	1.9	1.3	1.9	1.3	0.6

Supplementary Table 5. List of all H-2D[b]-binding peptides identified

Set I-T	Set T-I	Detected by both
AAPFDTVHI[a]	AAPRSFIFL[a]	AAITNKYQL[a]
AAPRNKHWL[a, b]	CQLGNFSIHM[a]	AAPRPPPKPM[a]
AGTRNIYYL[a, b]	FALANHLIKV[a]	AGVRNPQQHL[a]
AMGVNLTSM[a, b]	FCAVNPRFV[a, b]	AQIVNKHLI[a]
AQYGNILKHVM[a]	FGPVNHEEL[a, b]	ASVLNVNHI[a]
FAHTNIESL[a, b]	FINNNLHTQNL[a]	CSLQNKLVI[a]
FQHPNTDML[a, b]	FQIVNPHLL[a, b]	FAYEGRDYI[a]
GGVVNMYHM[a, b]	FSLHNPYNL[a, b]	FSFRNTQEVI[a]
IGIENIHYL[a, b]	FSPLNPVRVHI[a]	GGIQNVGHI[a]
IGPKNYEFL[a, b]	ISGVNRYYV[a, b]	GMIENGPFL[a]
KTVVNKDVF[a, b]	KAPDNRETL[a, b]	GQLSNGDHHF[a]
NSMVLFDHM[a]	KAPTNEFYA	GQLSNGDHHFM[a]
SAIHNFYDNI[a]	LCPSHFRETL[a]	ICPNNHEVHI[a]
SAIHNSTKV[a, b]	LSIQNYHLEC	KAIQNKDRAKVEF[a]
SAPRNFVENF[a]	LSLENGTHTL[a]	KALINADEL[a]
SAVVDKDFL[a]	RCHSNDSGNVL[a]	NSIRNLDTI[a]
SGIRNISFM[a, b]	SALQNAESDRL[a]	SAHQNYAEWL[a]
YALYNNWEHM[a]	SLGKNPTDAYL[a]	SAPENAVRM[a]
YQPYNKDWI[a, b]	SSIHNPTGRSYTI[a]	SMGKNPTDEYL[a]
YVHVNRDTL[a, b]	VGIRNTFLL[a, b]	SSLKNFQSCI[a]
	WCPRNPAVL[a, b]	TVPSVPSVPSVP
	YCYDNIHFM[a, b]	VSIEHQELM[a]
	YMPQNPCII[a, b]	YAGSNFPEHI[a]
	YQKAPTKEFYA	YGIRNSLLI[a]
		YSHRNQQMC

[a] Peptides with hydrophobic C-termini and analyzed in Fig. 4b–d for Set I-T and Set T-I, and in Fig. 4b for detected by both.

[b] Peptides with lengths of nine residues, hydrophobic C-termini and central anchor and analyzed in Fig. 5a, b.

Supplementary Table 6. List of all H-2K^b-binding peptides identified

Set I–T	Set T–I	Detected by both
FHAFYYVM[a, b]	ACVLYREL[a, b]	AAVKFHNL[a]
ICFKFDHL[a, b]	AICIFREL[a, b]	AAYEFTTL[a]
IMYDKHIQM[a]	CHYDFPVL[a, b]	AQFDFPSL[a]
ISYQFSNL[a, b]	ERPTYTNL[a, b]	ASYEFVQRL[a]
KIFEFKETL[a]	EVFDFRGMRL[a]	ATLVFHNL[a]
KVYNYNHL[a, b]	GAFDFPKL[a, b]	AWIHAAHV[a]
SGYIYHKL[a, b]	QIFRPDNF[a]	HGYTFANL[a]
SNYHFYSSI[a]	RNYEYLIRL[a]	HIYEFPQL[a]
SSYKFNHL[a, b]	RQYIFSKL[a, b]	HVYYFAHL[a]
STFIYNSM[a, b]	RVYYFNHI[a, b]	IAMEFNHL[a]
VGYRYETL[a, b]	SSFVFLNL[a, b]	INFDFPKL[a]
VNVDYSKL[a, b]	TIIIFHSL[a, b]	IQKTPQIQVY[a]
VQYKFSHL[a, b]	TNLVPYPRI[a]	ISFEFRSL[a]
VSYQFPKL[a, b]	TSVRFTQL[a, b]	ISFKFDHL[a]
	TVPSVPSVPSVP	LQYEFTKL[a]
	VAYKFPEL[a, b]	QSIEFSRL[a]
	VIVRFLTV[a, b]	QSYEFFHL[a]
	VNFVHTNL[a]	RAYLFAHV[a]
	VNQKFNNL[a, b]	RTYTYEKL[a]
	VSIQFYHL[a, b]	SGYDFENRL[a]
		SGYKFFSL[a]
		SHYDFGLRAL[a]
		SKYDFPKL[a]
		SSHSFPQL[a]
		STFVYNSM[a]
		STYKFFEV[a]
		TAYEFAKL[a]
		TNQDFIQRL[a]
		VGFDYKERL[a]
		VGPRYTNL[a]
		VNFEFPEF[a]
		VRYSHEKL[a]
		VSFDFHGRRM[a]
		VSFTYRYL[a]

[a] Peptides with hydrophobic C-termini and analyzed in Fig. 4b–d for Set I-T and Set T-I, and in Fig. 4b for detected by both.

[b] Peptides with lengths of eight residues, hydrophobic C-termini and central anchor and analyzed in Fig. 5a, b.

Supplementary Table 7. Comparison of frequencies of amino acids in H-2D^b-binding peptides

H2-D^b	Set I-T									Set T-I								
	P9	P8	P7	P6	P5	P4	P3	P2	P1	P9	P8	P7	P6	P5	P4	P3	P2	P1
D	0.0	0.0	0.0	0.0	0.0	0.0	28.6	0.0	0.0	0.0	0.0	0.0	20.0	0.0	0.0	0.0	0.0	0.0
E	0.0	0.0	0.0	7.1	0.0	0.0	14.3	0.0	0.0	0.0	0.0	0.0	0.0	0.0	0.0	20.0	10.0	0.0
K	7.1	0.0	0.0	7.1	0.0	21.4	0.0	7.1	0.0	10.0	0.0	0.0	0.0	0.0	0.0	0.0	0.0	0.0
R	0.0	0.0	0.0	21.4	0.0	7.1	0.0	0.0	0.0	0.0	0.0	0.0	20.0	0.0	20.0	10.0	0.0	0.0
H	0.0	0.0	21.4	7.1	0.0	0.0	14.3	14.3	0.0	0.0	0.0	0.0	10.0	0.0	10.0	20.0	0.0	0.0
L	0.0	0.0	0.0	0.0	0.0	7.1	0.0	0.0	50.0	0.0	0.0	10.0	0.0	0.0	0.0	0.0	20.0	60.0
I	14.3	0.0	28.6	0.0	0.0	28.6	0.0	0.0	14.3	10.0	0.0	20.0	0.0	0.0	10.0	0.0	10.0	10.0
V	0.0	7.1	14.3	28.6	0.0	7.1	0.0	7.1	7.1	10.0	0.0	0.0	40.0	0.0	0.0	0.0	10.0	20.0
M	0.0	7.1	0.0	0.0	0.0	7.1	0.0	7.1	21.4	0.0	10.0	0.0	0.0	0.0	0.0	0.0	0.0	10.0
F	14.3	0.0	0.0	0.0	0.0	0.0	0.0	14.3	7.1	40.0	0.0	0.0	0.0	0.0	0.0	10.0	20.0	0.0
Y	14.3	0.0	0.0	7.1	0.0	7.1	14.3	14.3	0.0	20.0	0.0	10.0	0.0	0.0	0.0	20.0	10.0	0.0
W	0.0	0.0	0.0	0.0	0.0	0.0	0.0	14.3	0.0	10.0	0.0	0.0	0.0	0.0	0.0	0.0	0.0	0.0
P	0.0	0.0	21.4	7.1	0.0	0.0	0.0	0.0	0.0	0.0	0.0	40.0	0.0	0.0	50.0	0.0	0.0	0.0
G	14.3	42.9	7.1	0.0	0.0	0.0	7.1	0.0	0.0	0.0	20.0	10.0	0.0	0.0	0.0	0.0	0.0	0.0
A	21.4	21.4	0.0	0.0	0.0	0.0	0.0	0.0	0.0	0.0	10.0	10.0	0.0	0.0	0.0	10.0	0.0	0.0
S	14.3	0.0	0.0	0.0	0.0	7.1	7.1	14.3	0.0	0.0	20.0	0.0	0.0	0.0	0.0	0.0	0.0	0.0
T	0.0	7.1	7.1	7.1	0.0	7.1	14.3	7.1	0.0	0.0	0.0	0.0	0.0	0.0	10.0	0.0	10.0	0.0
N	0.0	0.0	0.0	0.0	100.0	0.0	0.0	0.0	0.0	0.0	0.0	0.0	0.0	100.0	0.0	0.0	10.0	0.0
Q	0.0	14.3	0.0	7.1	0.0	0.0	0.0	0.0	0.0	0.0	10.0	0.0	10.0	0.0	0.0	0.0	0.0	0.0
C	0.0	0.0	0.0	0.0	0.0	0.0	0.0	0.0	0.0	0.0	30.0	0.0	0.0	0.0	0.0	10.0	0.0	0.0

Supplementary Table 8. Comparison of frequencies of amino acids in H-2K^b-binding peptides

H2-K^b	Set I-T								Set T-I							
	P8	P7	P6	P5	P4	P3	P2	P1	P8	P7	P6	P5	P4	P3	P2	P1
D	0.0	0.0	0.0	9.1	0.0	9.1	0.0	0.0	0.0	0.0	0.0	14.3	0.0	0.0	0.0	0.0
E	0.0	0.0	0.0	0.0	0.0	9.1	0.0	0.0	7.1	0.0	0.0	0.0	0.0	0.0	21.4	0.0
K	9.1	0.0	0.0	27.3	0.0	0.0	27.3	0.0	0.0	0.0	0.0	14.3	0.0	0.0	14.3	0.0
R	0.0	0.0	0.0	9.1	0.0	0.0	0.0	0.0	14.3	7.1	0.0	14.3	0.0	14.3	0.0	0.0
H	0.0	9.1	0.0	0.0	0.0	9.1	36.4	0.0	0.0	7.1	0.0	0.0	0.0	7.1	14.3	0.0
L	0.0	0.0	0.0	0.0	0.0	0.0	0.0	81.8	0.0	0.0	0.0	7.1	0.0	14.3	0.0	85.7
I	18.2	0.0	0.0	18.2	0.0	0.0	0.0	0.0	0.0	21.4	14.3	21.4	0.0	0.0	0.0	7.1
V	36.4	9.1	9.1	0.0	0.0	0.0	9.1	0.0	28.6	7.1	21.4	7.1	0.0	0.0	7.1	7.1
M	0.0	0.0	0.0	0.0	0.0	0.0	0.0	18.2	0.0	0.0	0.0	0.0	0.0	0.0	0.0	0.0
F	9.1	0.0	18.2	9.1	45.5	0.0	0.0	0.0	0.0	0.0	14.3	0.0	85.7	0.0	0.0	0.0
Y	0.0	0.0	63.6	0.0	54.5	9.1	0.0	0.0	0.0	0.0	28.6	7.1	14.3	7.1	0.0	0.0
W	0.0	0.0	0.0	0.0	0.0	0.0	0.0	0.0	0.0	0.0	0.0	0.0	0.0	0.0	0.0	0.0
P	0.0	0.0	0.0	0.0	0.0	9.1	0.0	0.0	0.0	0.0	7.1	0.0	0.0	21.4	0.0	0.0
G	0.0	18.2	0.0	0.0	0.0	0.0	0.0	0.0	7.1	0.0	0.0	0.0	0.0	0.0	0.0	0.0
A	0.0	0.0	9.1	0.0	0.0	0.0	0.0	0.0	14.3	14.3	0.0	0.0	0.0	0.0	0.0	0.0
S	27.3	27.3	0.0	0.0	0.0	27.3	9.1	0.0	7.1	21.4	0.0	0.0	0.0	7.1	7.1	0.0
T	0.0	9.1	0.0	0.0	0.0	0.0	9.1	0.0	14.3	0.0	0.0	7.1	0.0	14.3	7.1	0.0
N	0.0	9.1	0.0	9.1	0.0	27.3	9.1	0.0	0.0	7.1	0.0	0.0	0.0	14.3	21.4	0.0
Q	0.0	9.1	0.0	18.2	0.0	0.0	0.0	0.0	0.0	7.1	7.1	7.1	0.0	0.0	7.1	0.0

Supplementary Table 9. Summary of variant peptides examined in this study and their activities

Motif	Peptide	TCR	Agonist activity	Dulling	Positive selection (FTOC)
tCP	OVA-P6	OT-I	-	+	+
	OVA-D7	OT-I	-	+	+
	OVA-E7	OT-I	-	+	+
	gp33-P6	P14	+	++	Not tested
	NP68-P6	F5	-	-	Not tested
iCP	OVA-N6	OT-I	+++	+++	-
	OVA-S6	OT-I	+	++	+
	OVA-H7	OT-I	+++	+++	-
	gp33-K6	P14	-	-	Not tested
	NP68-K6	F5	-	-	Not tested

For the agonist activity and dulling assay, peptides showing >70%, 40–70%, 10–40%, and <10% of the responses induced by the antigenic original peptides at 1 μM were assigned +++, ++, +, and -, respectively. Peptides were assigned + for the positive selection when the average numbers of $V\alpha2^{high}$ CD8SP thymocytes generated in FTOC was significantly ($P<0.05$) higher than those in the control unrelated peptides.
